# Separation Techniques and Circular Economy

**DOI:** 10.3390/membranes13090778

**Published:** 2023-09-04

**Authors:** Da-Qi Cao

**Affiliations:** 1 Sino-Dutch R&D Centre for Future Wastewater Treatment Technologies/Key Laboratory of Urban Stormwater System and Water Environment, Beijing University of Civil Engineering and Architecture, Beijing 100044, China; caodaqi18@163.com; Tel.: +86-(0)-10-6832-2123; Fax: +86-(0)-10-6832-2128; 2 Yau Mathematical Sciences Center, Tsinghua University, Beijing 100084, China

Efficient separation techniques play an important role in the process of resource recovery, and these techniques include physical, chemical, physicochemical, and/or biological methods that are selected for their low cost and low energy consumption and for being free of secondary pollution. Additionally, the highest possible value added of the separated products is obtained to enhance the economy. This current Special Issue titled “Separation Techniques and Circular Economy” in *Membranes* presents the novel separation technologies involving the recovery of extracellular polymeric substances (EPSs) [1], calcium alginate (Ca-Alg) [2] in wastewater treatment, plasmid DNA in gene therapy [3], lactose in dairy industry [4], lactic acid (LA) in the fermented broth of kitchen waste [5,6], phenylalanine losses in neutralization dialysis (ND) [7], the removal of secondary effluent organic matter (EfOM) [8], and acetaldehyde (CH_3_CHO) in the atmosphere [9].

For the recycling of EPSs from excess sludge in wastewater treatment plants (WWTPs), the potential of nanofiber membranes for use in effective concentration and the recycling of EPSs via membrane separation was indicated [1]. Electrospun nanofiber membranes (ENMs) were fabricated using polyvinylidene fluoride (PVDF) and used for the recovery of EPSs extracted from the excess sludge using the cation exchange resin (CER) method. The fabricated ENM containing 14 wt.% PVDF showed excellent properties, with a high average water flux (376.8 L/(m^2^·h)) and an excellent EPS recovery rate (94.1%) in the dead-end filtration of a 1.0 g/L EPS solution at 20 kPa. The ENMs displayed excellent mechanical strength, antifouling properties, and high reusability after five recycles. The filtration pressure had a negligible effect on the average EPS recovery rate and water flux. The novel dead-end filtration with an EPS filter cake on the ENM surface was effective in removing heavy-metal ions, with the removal rates of Pb^2+^, Cu^2+^, and Cr^6+^ being 89.5%, 73.5%, and 74.6%, respectively.

Ca-Alg is a novel target product for recovering alginate from aerobic granular sludge. A novel production method, where Ca-Alg was formed in a sodium alginate (SA) feed solution (FS) and concentrated via forward osmosis (FO) with Ca^2+^ reverse osmosis using a draw solution of CaCl_2_, was proposed [2]. An abnormal reverse solute diffusion was observed, with the average reverse solute flux (RSF) decreasing with increasing CaCl_2_ concentrations, while the average RSF increased with increasing alginate concentrations. The RSF of Ca^2+^ in FS decreased continuously as the FO progressed using 1.0 g/L SA as the FS, while it increased initially and later decreased using 2.0 and 3.0 g/L SA as the FS. These results were attributed to the Ca-Alg recovery production (CARP) that formed on the FO membrane's surface on the feed side, and the percentage of Ca^2+^ in CARP relative to total Ca^2+^ reverse osmosis reached 36.28%. Scanning electron microscopy and energy dispersive spectroscopy also verified CARP's existence and its Ca^2+^ content. The thin-film composite FO membrane with a supporting polysulfone electrospinning nanofiber membrane layer showed high water flux and the RSF of Ca^2+^, which was proposed as a novel FO membrane for Ca-Alg production via the FO process with Ca^2+^ reverse diffusion. Four mechanisms, including the molecular sieve role, the electrification of colloids, the osmotic pressure of ions in CARP, and the FO membrane structure, were proposed to control Ca-Alg production.

Plasmid DNA is used as a vector for gene therapy and DNA vaccination. Because of its circular structure, a significant deformation occurs during filtration and easily permeates the membrane, hindering the selection of separation membranes based on molecular weight. Affinity microfiltration (MF) and plasmid DNA purification were applied [3]. *A*-Fe_2_O_3_ with an isoelectric point of approximately 8 and a particle size of 0.5 μm was selected as the ligand for the two-stage affinity MF of plasmid DNA. In the first stage of MF, the experiment was conducted at a pH of 5, and a cake of *α*-Fe_2_O_3_ with bound plasmid DNA was obtained. Next, liquid permeation (pH 9 and 10) through the cake was performed to elute the bound plasmid DNA. Plasmid DNA was eluted during the early phase of liquid permeation at a pH of 10. Furthermore, agarose gel analysis confirmed the usefulness of the two-stage affinity MF method with the adsorption and desorption for plasmid DNA purification.

The lactose in the dairy industry is not produced profitably. Nanofiltration (NF) concentration has shown to be a good choice since partial demineralization can be realized in parallel. Ten commercial polymer NF membranes were systematically studied in detail for their suitability to concentrate lactose, with the proviso of high flux and high to complete rejection [4]. Preliminary trials were conducted using flat-sheet membranes and a lactose model solution, and the influence of transmembrane pressure, temperature, and lactose concentration was studied. Finally, the results were evaluated using spiral wound modules and real industrial whey permeate. Membrane screening is essential since no correlation between molecular weight cut-off and permeate flow could be found. The conclusions found for the lactose model solution were in good agreement with the whey permeate, but as ions contribute to the osmotic pressure of the feed, the deviations increase during the concentration process since ions are also partly retained.

Lactic acid (LA) is an important chemical material facing rapid demand in recent years. The oriented fermentation of kitchen waste is a promising route for economic LA production. However, the purification of the LA from the broth is still troublesome when considering the economy. The purification performance of MF membrane technology for a fermentation broth from kitchen waste was investigated [5]. The results indicated that the MF process could be a desirable broth purification process to some extent, and it is promising in actual application. Under the optimum pressure of 100 kPa, a pH of 6.0, and a backflushing mode with deionized water for 3 min, the best performance was achieved, with chroma removal, turbidity removal, protein removal, and total sugar removal efficiencies of 60, 92.8, 57.64, and 32.93%, respectively. On the other hand, the refinement of LA from fermentation broth is also a spiny issue. The performance of the ultrafiltration (UF) process for the refinement of LA from the pre-microfiltered broth of kitchen waste fermentation was investigated [6]. With a 50 kDa polyethersulfone membrane, under the optimum pressure of 120 kPa, the pH of 6.0, and the backflushing mode with the deionized water for 3 min, the best performance was achieved with respect to the chroma removal efficiency, turbidity removal efficiency, protein removal efficiency, and total sugar removal efficiency of 54.3%, 89.8%, 71.7% and 58.5%, respectively, and the LA recovery efficiency was 93.6%. The results indicated that the UF process could further effectively refine the pre-microfiltered broth of kitchen waste fermentation, and the combination of MF and UF process is ideal for achieving desirable LA refinement performance.

Amino acid (phenylalanine (Phe)) and mineral salt (NaCl) solution separation can be carried out using neutralization dialysis (ND) [7]. A non-steady state mathematical model of the Phe and NaCl solution's separation via ND in a batch mode is proposed. The model takes into account the characteristics of membranes (thickness, ion-exchange capacity, and conductivity) and solutions (concentration and composition). Compared to previously developed models, the new model considers the local equilibrium of Phe protolysis reactions in solutions and membranes and the transport of all phenylalanine forms (positively and negatively charged zwitterionic) through membranes. A series of experiments on the ND demineralization of the NaCl and Phe mixed solution was carried out. In order to minimize Phe losses, the solution pH in the desalination compartment was controlled by changing the concentrations of the solutions in the acid and alkali compartments of the ND cell. The validity of the model was verified by carrying out a comparison of simulated and experimental time dependencies with respect to the solution's electrical conductivity and pH, as well as the concentration of Na^+^, Cl^−^ ions, and Phe species in the desalination compartment. Based on the simulation's results, the role of Phe transport mechanisms in the losses of this amino acid during ND was discussed. In the experiments carried out, the demineralization rate reached 90%, accompanied by minimal Phe losses of about 16%. Modeling predicts a steep increase in Phe losses when the demineralization rate is higher than 95%. Nevertheless, simulations show that it is possible to achieve a highly demineralized solution (by 99.9%) with Phe losses amounting to 42%.

The reuse of wastewater has been identified as an important initiative for the sustainable development of the environment; thus, the removal of secondary EfOM to ensure the safety of reused wastewater is the key step and a subject of extensive research. Al_2_(SO_4_)_3_ and anionic polyacrylamide were selected as the coagulant and flocculant, respectively, for the treatment of secondary effluents from a food-processing industry WWTP to meet the standard regulatory specifications for water reuse [8]. This process is efficient and economically viable for EfOM removal to realize food-processing wastewater reuse. The removal efficiencies of chemical oxygen demand (COD), components with UV_254_, and specific ultraviolet absorbance were 44.61%, 25.13%, and 9.13%, respectively, with an associated reduction in chroma and turbidity. The fluorescence intensities (Fmax) of two humic-like components were reduced during coagulation, and the microbial humic-like components of EfOM had a better removal efficiency because of a higher Log*K*_m_ value of 4.12. Fourier-transform infrared spectroscopy showed that Al_2_(SO_4_)_3_ could remove the protein fraction of the soluble microbial products (SMPs) of EfOM by forming a loose SMP protein complex with enhanced hydrophobicity. Furthermore, flocculation reduced the aromaticity of secondary effluent. The cost of the proposed secondary effluent treatment was 0.034 CNY t^−1^ %COD^−1^.

CH_3_CHO in the atmosphere is associated with adverse health effects. Among the various options for use in removing CH_3_CHO, adsorption is often employed because of its convenient application and economical processes, particularly when using activated carbon. The surface of activated carbon has been modified with amines to remove CH_3_CHO from the atmosphere via adsorption. However, these materials are toxic and can have harmful effects on humans when the modified activated carbon is used in air-purifier filters. Therefore, customized bead-type activated carbon (BAC) with surface modification options via amination was evaluated for removing CH_3_CHO [9]. Various amounts of non-toxic piperazine or piperazine/nitric acid were used in amination. Chemical and physical analyses of the surface-modified BAC samples were performed using Brunauer–Emmett–Teller measurements, elemental analyses, and Fourier-transform infrared and X-ray photoelectron spectroscopy. The chemical structures on the surfaces of the modified BACs were analyzed in detail using X-ray absorption spectroscopy. The amine and carboxylic acid groups on the surfaces of the modified BACs are critical in CH_3_CHO adsorption. Notably, piperazine amination decreased the pore size and volume of the modified BAC, but piperazine/nitric acid impregnation maintained the pore size and volume of the modified BAC. In terms of CH_3_CHO adsorption, piperazine/nitric acid impregnation resulted in superior performances, with greater chemical adsorption. The linkages between the amine and carboxylic acid groups may function differently in piperazine amination and piperazine/nitric acid treatment.

Overall, the Special Issue presents helpful contributions to the recovery of biopolymers (EPS, Ca-Alg, and DNA) or micromolecules (lactose, LA, and phenylalanine) and the removal of EfOM and CH_3_CHO, in which various separation techniques such as ENM separation, MF, UF, NF, FO, ND, flocculation, and adsorption are used. However, in practice, separation techniques that are involved in the circular economy include numerous methods, and these are dependent on the recycled or separated products. In conclusion, the editors appreciate the authors’ and reviewers’ valuable contributions to this Special Issue.
